# Application of physician-modified fenestrated stent graft in urgent endovascular repair of abdominal aortic aneurysm with hostile neck anatomy: Case report

**DOI:** 10.1097/MD.0000000000005455

**Published:** 2016-11-18

**Authors:** Rong Zeng, Wei Ye, Changwei Liu, Xuan Wang, Xiaojun Song, Leng Ni, Bao Liu, Yongjun Li, Yuehong Zheng

**Affiliations:** a Department of Vascular Surgery; b Department of Radiology, Peking Union Medical College Hospital, Chinese Academy of Medical Sciences & Peking Union Medical College, Beijing, China.

**Keywords:** abdominal aortic aneurysm, case report, endovascular aneurysm repair, fenestration, physician-modified fenestration, scallop

## Abstract

**Background:**

This study aimed to evaluate the feasibility and effectiveness of the Gore Excluder aortic stent graft (WL Gore & Associates, Inc., Flagstaff, AZ) using the C3 Delivery System after physician modification of fenestration for the urgent treatment of patients with abdominal aortic aneurysm showing hostile neck anatomy.

**Case summary:**

Three urgent cases of abdominal aortic aneurysm with hostile neck anatomy symptom with abdominal pain were reported. The same fenestration method was applied to align the target superior mesenteric artery and bilateral renal arteries with 1 scallop and 2 fenestrations, followed by the reconstruction of the target artery using a bare-metal stent or stent graft. Balloon-assisted positioning and image fusion technology were intraoperatively applied to assist the accurate release of the stent graft body. The follow-up periods for all cases exceeded 6 months, showing smooth circulation in the target arteries with no endoleaks.

**Conclusion:**

In the absence of other available treatment methods, it is feasible to use a stent graft with physician-modified fenestration for the urgent endovascular repair of abdominal aortic aneurysm with hostile neck anatomy. However, this procedure's long-term efficacy needs to be further investigated.

## 1 Introduction

Since Parodi et al^[1]^ first applied endovascular aneurysm repair to successfully treat abdominal aortic aneurysms in 1991, it has gradually become the preferred alternative option over open surgery because of its relatively small surgical trauma and quick postoperative recovery.^[2]^ However, for abdominal aortic aneurysm with complex anatomy in the aneurismal neck, which cannot tolerate open surgery, conventional commercial stents cannot be easily applied, and the combination of chimney/fenestration and other special technologies is required to complete the aneurysm repair surgery.^[3–5]^ Despite the ability to apply a commercial fenestrated stent to the treatment of abdominal aortic aneurysm with hostile neck anatomy,^[6]^ it is limited to a production cycle of 6 to 8 weeks and is not available for emergent surgery or limited operation.^[7]^ Hostile neck anatomy has 1 or more following features: neck length <15 mm, neck angulation >60°, proximal neck thrombus or calcification covering >50% of the circumference of the aortic diameter, and a reverse taper morphology. In this study, 3 cases of patients with aortic aneurysm showing hostile neck anatomy requiring urgent treatment were provided with the C3 Delivery System Excluder stent (W.L. Gore & Associates, Inc., Flagstaff, AZ). Before implantation, the scallops and fenestrations were created after precise positioning for the endovascular repair of the aneurysms; meanwhile, the 3 target arteries of the superior mesenteric artery and bilateral renal arteries were reconstructed. The follow-up periods of all 3 cases were exceeded 6 months; in each case, smooth circulation in the main and branch stent arteries with no endoleaks was confirmed. These results suggest that the application of this method in a limited operation for abdominal aortic aneurysm with hostile neck anatomy is feasible.

The 3 patients in this study were regarded as not tolerating or not accepting open surgery during the clinical assessment. They were informed the benefits and risks of off-label endovascular repair and signed consents.

## 2 Case report

### 2.1 Case 1

**Figure 1:**
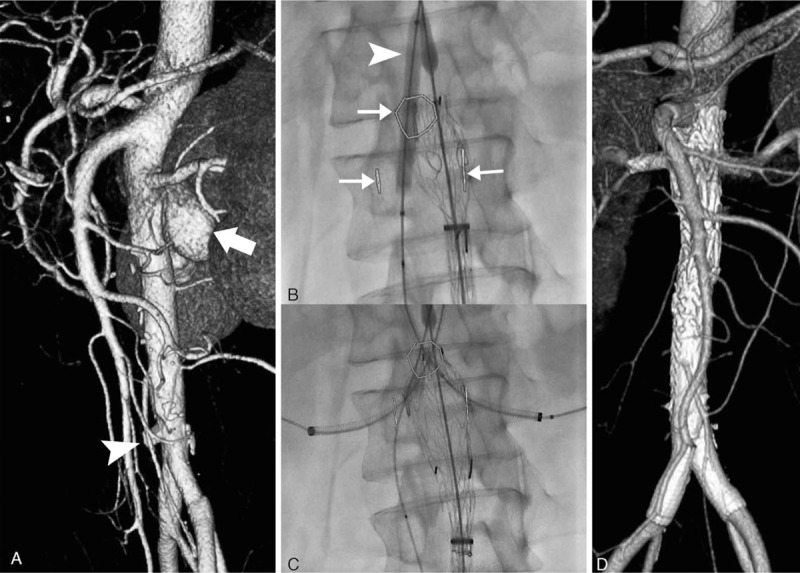
Preoperative CTA (A) showed an abdominal aortic pseudoaneurysm located in the posterior wall of the abdominal aorta at the level of the renal artery (white arrow in A) and an aortic pseudoaneurysm located in the anterior wall of the end of the abdominal aorta (white arrowhead in A). A balloon catheter (EverCross, ev3 Inc.) was intraoperatively (B, C) placed in the superior mesenteric artery (white arrowhead in B) to assist with the release of the stent graft body so that the “V”-shaped scallop could be aligned to the opening of the mesenteric artery. The target arteries’ openings were labeled using image-fusion technology (white arrows in B) so that they were always visible in fluoroscopy imaging. Using the approach from the upper-limb arteries, the bilateral renal arteries could be selected through the fenestrations (C). The CTA follow-up at 6 months after surgery showed that (D) the circulation of the superior mesenteric artery and bilateral renal arteries was smooth and that the pseudoaneurysm was isolated completely, with no endoleak. CTA = computed tomographic angiography.

A male patient, aged 43 years, had suffered from abdominal pain accompanied by a high fever for 3 months and was diagnosed with infectious abdominal aortic aneurysms. The blood culture from another hospital revealed *Klebsiella pneumoniae*. Before being transferred to our hospital, he had received intravenous antibiotic that was effective against the bacteria for 6 weeks, with a normal body temperature and hemogram for 4 weeks, but the abdominal pain was not completely relieved. The patient had undergone laparotomy a year before for drainage around the pancreas due to acute severe pancreatitis. Preoperative computed tomographic angiography (CTA) (Fig. 1A) suggested 2 abdominal aortic pseudoaneurysms, which were located in the posterior wall of the abdominal aorta and the anterior wall of the end of the abdominal aorta at the level of the renal artery.

**Figure 2:**
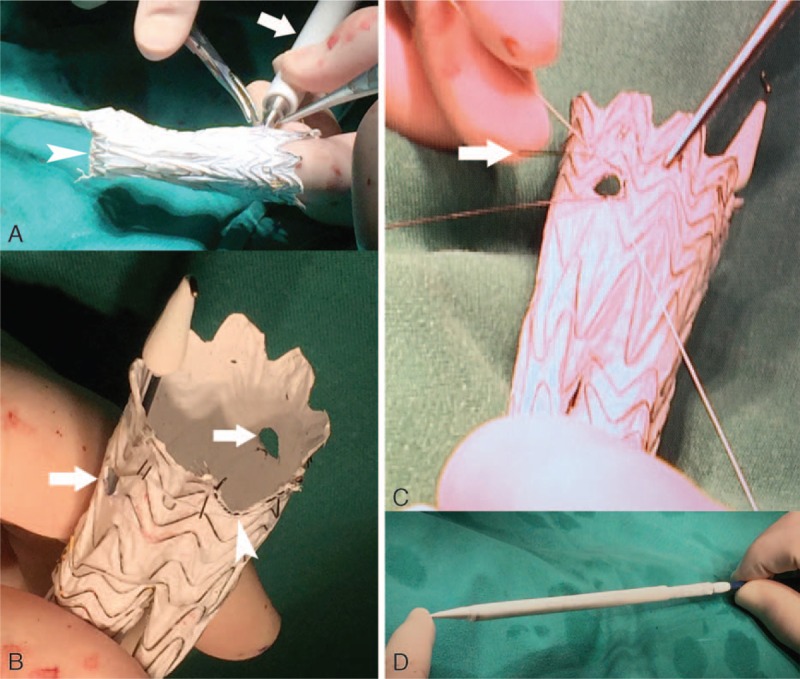
Preparation of the main body of the stent graft. The short iliac leg branch of the main stent graft body was sutured using CV-7 ePTFE sutures (Gore-tex) (white arrowhead in A). A fenestration at the predetermined site was created on the stent graft body using a vascular puncher (white arrow in A) for the right renal artery. The fenestrations were performed on the stent graft body for the bilateral renal arteries (white arrows in B), and a “V”-shaped scallop was created for the superior mesenteric artery (white arrowhead in B). The head (white arrow in C) of the developing portion of the 0.014-inch guiding wire (ASAHI INTECC, Thailand) was sutured on the edge of the scallop and fenestration with a CV-7 ePTFE suture (Gore-tex) for reinforcement, while allowing the fenestration portion to be visible in fluoroscopy imaging. (D) The stent graft body was reassembled into the truncated DrySeal sheath (WL Gore & Associates, Inc., Flagstaff, AZ). ePTFE = expanded polytetrafluoroethylene.

On the third day after admission, surgical treatment was performed. A total of 3 arterial approaches for the upper limbs were established, and an arterial approach was established for each lower limb. The preparation of the main body of the stent graft (Fig. 2) was completed in the sterile field of the operating table. The positions of the scallop and fenestration in the stent graft body were determined on the basis of the preoperative CTA using the clock-face method. After the stent graft body was released in vitro, the short iliac leg branch was sutured (Fig. 2A). Using a 5.2-mm single-use vascular puncher (Cardio vision supercut; International Biophysics, Austin, TX), 2 fenestrations were performed on the stent graft body (Fig. 2A), and a scallop was created at the proximal end of the stent graft body (Fig. 2B). Then, the head of the developing portion of the guiding Regalia XS 1.0 wire (ASAHI INTECC, Tambol Bangkadi, Thailand) was sutured in the scallop and fenestrations (Fig. 2C), playing the role of reinforcing and developing during the surgery. The stent graft body was released with the assistance of positioning by balloon of the superior mesenteric artery and using the intraoperative image fusion technique (Fig. 1B, C). The bilateral renal arteries were accessed through the upper limb approaches. After confirming that the renal artery had been selected for entry, a CODA balloon (Cook Medical Inc., Bloomington, IN) was expanded at the proximal sealing zone of the stent graft. The bilateral renal arteries and the superior mesenteric artery were reconstructed with the balloon-expandable stent. Because the diameter of the abdominal aorta in the patient was small, the short iliac leg was sutured, and 2 stent grafts (Viabahn, W.L. Gore & Associates, Inc., Flagstaff, AZ) were placed in the long iliac leg in parallel. Both iliac arteries were reconstructed, and the pseudoaneurysm at the end of the abdominal aorta was isolated. The CTA follow-up 6 months after the surgery showed that (Fig. 1D) the blood flow in the bilateral renal and superior mesenteric arteries as well as in the bilateral iliac arteries was smooth and that the pseudoaneurysm was completely isolated, with no endoleaks.

### 2.2 Case 2

**Figure 3:**
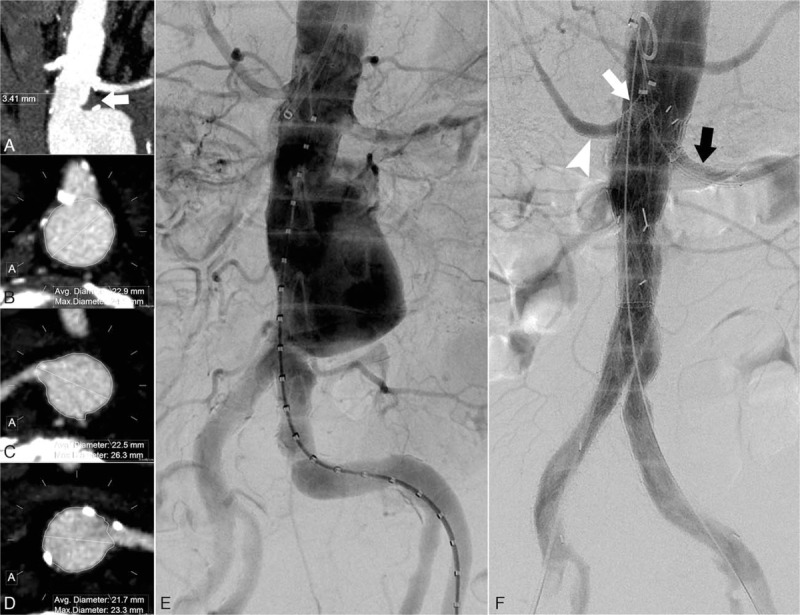
The preoperative CTA. (A) Imminent rupture of the abdominal aortic aneurysm, with an aneurismal neck length of 3.4 mm and exudation near the aneurismal neck (white arrow in A). Preoperative measurement of the positions of the superior mesenteric artery (B) and bilateral renal arteries (C, D) was conducted by means of clock-face orientation. Preoperative digital subtraction angiography (DSA) (E) revealed an abdominal aortic aneurysm with hostile neck anatomy. Endovascular repair of the abdominal aortic aneurysms was carried out with the reconstruction of the mesenteric artery using a balloon-expandable stent (Scuba, InvatecS.pA, Italy) (white arrow in F). After the reconstruction of the right renal artery using a balloon-expandable stent (Paramount Mini GPS; ev3 Inc.) (white arrowhead in Fin F), a dissection of the left renal artery was found. The stent graft (Viabahn; WL Gore & Associates, Inc., Flagstaff, AZ) and self-expanding stent (Zilver, Cook Ireland Ltd., Limerick, Ireland) were used to extend into the left renal artery (black arrow in F). The postoperative DSA (F) showed that the blood flow of bilateral renal arteries was smooth. CTA = computed tomographic angiography, DSA = digital subtraction angiography.

A male patient, aged 64 years, had suffered an abdominal aortic aneurysm for 3 years, with an aneurysm diameter of 3.5 cm at diagnosis and with an irregular hospital referral. The main complaint was persistent abdominal pain for 20 days, and the pain was aggravated the day before the patient visited our hospital. A pulsation mass was found at epigastrium, with tenderness. The diagnosis was imminent rupture of an abdominal aortic aneurysm. The patient had a history of hypertension for 10 years, an appendectomy 47 years before, and a right hepatectomy with cholecystectomy due to liver cancer 23 years before. In addition, the patient had been in the decompensated stage of chronic renal insufficiency for 3 years, with a creatinine level of 170 to 200 μmol/L in the last 6 months. The preoperative CTA revealed an abdominal aortic aneurysm with a maximum diameter of 5.0 cm, an aneurismal neck length of 3.4 mm, and exudation outside the artery wall at the aneurismal neck (Fig. 3A).

In the second day after admission, the patient underwent endovascular repair surgery for abdominal aortic aneurysm, with the positioning of fenestrations using the same clock-face method (Fig. 3B–D). The intraoperative procedure was similar to that of Case 1. Because of a new dissection in the left renal artery, a stent graft (Viabahn, W.L. Gore & Associates, Inc., Flagstaff, AZ) and a self-expanding stent (Zilver, Cook Ireland Ltd., Limerick, Ireland) were intraoperatively placed in the renal artery to guarantee the left renal blood supply (Fig. 3E, F). The bilateral iliac arteries were reconstructed using stent grafts (contralateral leg endoprosthesis; W.L. Gore & Associates, Inc., Flagstaff, AZ). Postoperative recovery was satisfactory, and renal function creatinine was maintained at 200 to 240 μmol/L at 6 months after the surgery. To avoid damage to the renal function of the patient, color ultrasound was used at follow-up. The color ultrasound follow-up at 6 months after surgery confirmed that the blood flow of the bilateral renal arteries and the superior mesenteric artery was smooth, with no endoleaks of the aneurysm.

### 2.3 Case 3

**Figure 4:**
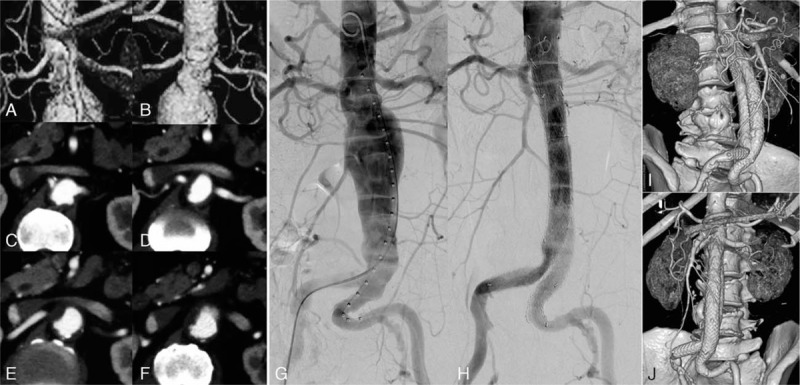
Preoperative CTA reconstruction (A, B) revealed an abdominal aortic aneurysm with hostile neck anatomy. Continuous cross-section scanning (C–F) revealed an aneurismal neck mural thrombus covering 50% of the circumference and an imminent rupture of the aneurysm in the aneurismal neck area (F). Intraoperative DSA (G, H) revealed that the abdominal aortic aneurysm was well repaired with a fenestrated stent graft. The 3 target arterial branches were reconstructed using a balloon-expandable stent (Scuba, InvatecS.r.l., Italy) for the superior mesenteric artery, and 2 stent grafts (Viabahn; W.L. Gore & Associates, Inc., Flagstaff, AZ) were used for the reconstruction of the bilateral renal arteries. The CTA follow-up at 6 months after surgery (I, J) showed smooth flow in the target arteries, with no aneurysm endoleaks. CTA = computed tomographic angiography, DSA = digital subtraction angiography.

A male patient, aged 66 years, had suffered continuous severe abdominal pain for 3 days and was transferred to our hospital with the diagnosis of abdominal aortic aneurysm revealed by a CTA examination. The patient had experienced complicated hypertension for 20 years, coronary heart disease for 5 years, coronary stent implantation 2 years prior, and diabetes for 3 years, psoriasis for 30 years, and gastroduodenal resection due to gastric ulcer 20 years before. Preoperative CTA (Fig. 4A–F) revealed a juxtarenal abdominal aortic aneurysm, with a 1/2 circumference mural thrombus in the aneurismal neck. After antihypertensive treatment, abdominal pain could not be completely relieved. In the second day after admission, a similar method of fenestration was applied for the endovascular repair of the abdominal aortic aneurysm (Fig. 4G, H), with the reconstruction of the superior mesenteric artery using a balloon-expandable stent and the reconstruction of the bilateral renal arteries using a stent graft (Viabahn, W.L. Gore & Associates, Inc., Flagstaff, AZ). The patient was successfully discharged after the surgery. The CTA follow-up at 6 months (Fig. 4I, J) after the surgery showed that the circulation of the superior mesenteric artery and bilateral renal arteries was smooth, with no endoleaks.

## 3 Discussion

The Cook Zenith aortic endograft (Cook Medical Inc., Bloomington, IN) is the only commercial stent system for the treatment of juxtarenal abdominal aortic aneurysm to have been approved by the US Food and Drug Administration (FDA), and its effectiveness and long-term efficacy have been verified,^[8]^ but because of concern about its production cycle, it is difficult to use this stent in emergent surgery. The C3 release system of the Gore Excluder aortic stent graft (W.L. Gore & Associates, Inc., Flagstaff, AZ) allows the repeatable release of the anchoring portion at the proximal end of the stent graft body so that it is possible to be modified by physicians and to be recoverable and adjustable until optimal positioning is achieved.

The selection for the position of fenestration in the stent graft requires full consideration of the position of the target vessel and the metal structure of the stent itself. The fenestration position in the stent body is commonly determined by the clock-face method. Previously, existing studies had described in detail how to carry out physician modification and fenestration in a stent graft body.^[9]^ In this study, in addition to similar fenestration in the stent graft body, a V-shaped scallop was also created in the anchoring structure region so that the bilateral renal arteries and the mesenteric artery could be aligned. The anchoring structure of the Gore Excluder was designed in a range of 9 mm around the proximal end of the stent graft. The proximal end of the stent within this range could fit in a “V”-shaped scallop, but it might destroy the anchoring structure, so a balloon-expandable stent was placed in the site of the scallop, which would not only ensure that the anchoring structure was sufficiently supported after the scallop was created but also ensure the blood supply of the target artery. To avoid damaging the anchoring structure, the structure of more than 2 scallops is not recommended in the proximal end of the stent graft. When the target artery was the bilateral renal artery, the fenestration position needed to be designed in a position range of 9 to 40 mm (applied for the 23-, 26-, and 28-mm stents), in a position range of 9 to 50 mm (applied for the 31-mm stent), or in a position range of 9 to 60 mm (applied for the 35-mm stent).

The tectorial membrane structure of the Excluder stent is expanded polytetrafluoroethylene (ePTFE) material. On the basis of the diameter of the target artery, we chose a 5.2-mm single-use vascular puncher to conduct fenestration in the stent graft body (Fig. 2A) so that the shape of the fenestration was more regular and the chance of endoleak was reduced. The diameter of the puncher is slightly smaller than the diameter of the target vessel and the diameter of the stent for the target artery reconstruction so that the anchoring rigor can be further enhanced. The head portion of the guiding wire (Regalia XS 1.0 wire; ASAHI INTECC, Thailand) for developing was then sutured at the site of the fenestration for reinforcement (Fig. 2B, C). The fenestration position can be observed in the fluoroscopy image.

The accurate alignment of the fenestration and scallop to the target artery is key for the success of fenestrated endovascular aortic aneurysm repair (fEVAR). Image fusion technology and balloon-assisted positioning technology had been applied to the release process of the stent body. The opening of the superior mesenteric artery and the bilateral renal arteries were labeled in the preoperative CTA imaging, while image fusion was performed for the preoperative CTA and intraoperative C-arm cone-beam computed tomography, so that the exact position of the target artery could be observed intraoperatively.^[10]^ Before the release of the stent graft body, a balloon was preset in the superior mesenteric artery, so that the “V” scallop could be accurately aligned with the superior mesenteric artery.

We did not preset the guiding wire passing through the fenestration portion; thus, the diameter for sheath delivery was minimized. The renal arteries were selected by the upper limb approach so that the target artery could be selected when the stent graft body was not completely released, allowing the stent graft body to be adjusted more easily.

Our successful practice on these cases with impending or contained ruptured abdominal aortic aneurysm with hostile neck anatomy suggested that physician-modified fenestrated stent grafts with the C3 Delivery System may have a future role in the compassionate treatment, when commercial stent grafts are not available.

## 4 Conclusion

In the absence of other available treatment methods, it is feasible to use a stent graft with C3 release system with physician-modified fenestration for the urgent endovascular repair of complex abdominal aortic aneurysms. However, this procedure's long-term efficacy needs to be further investigated.
